# COVID-19 infections among Iraqi school students: Severity, types, and symptoms

**DOI:** 10.25122/jml-2023-0256

**Published:** 2023-10

**Authors:** Saad Hantoosh

**Affiliations:** 1 Department of Science, Open Educational College, Ministry of Education, Samawa, Iraq

**Keywords:** COVID-19, asymptomatic, pre-symptomatic, symptomatic

## Abstract

The prevalence of COVID-19 infections among school students has become a significant and ongoing concern. This study aimed to assess the severity and types of COVID-19 cases and associated symptoms among school students in Iraq. A comprehensive study was conducted by the Public Health Directorate of AL-Muthanna Governorate from November 29, 2020, to February 12, 2021, utilizing RT-PCR-based COVID-19 surveys. The survey included 9,357 students (4,261 male and 5,096 female) and 83 schools. The retrospective analysis of the survey records indicated that male and female students had a mean age of 9.2±1.16 years with notably higher rates of asymptomatic infections than older students. Male students showed lower odds of asymptomatic infection but higher odds of symptomatic and pre-symptomatic infections compared to their female peers, particularly among elementary students. Fever, abdominal pain, diarrhea, loss of taste and smell, shortness of breath, and muscle pain were significantly associated with COVID-19 infection. Sneezing was significantly associated with a lack of infection. Home nursing by parents and self-care practices have proven to be highly effective in controlling COVID-19 infection among children. These findings highlight the need for age- and gender-specific considerations in COVID-19 prevention and management strategies in schools.

## INTRODUCTION

Since the early 2000s, three novel coronaviruses of zoonotic origin have emerged: severe acute respiratory syndrome coronavirus (SARS-CoV-1), Middle East respiratory syndrome coronavirus (MERS-CoV), and severe acute respiratory syndrome coronavirus 2 (SARS-CoV-2) responsible for the coronavirus disease 2019 (COVID-19) [1]. The COVID-19 pandemic has brought unprecedented challenges to the global community, affecting millions of lives and livelihoods. The impact of COVID-19 has been profound, with a devastating loss of life and prolonged health consequences [2]. The global infection fatality rates (IFRs) of COVID-19 exhibit significant heterogeneity. While some countries were struggling with waves of severe cases, others were reporting relatively low IFRs [3-5]. The symptoms and severity of COVID-19 infection can vary widely from person to person, with substantial variations across continents and countries. Factors such as age, underlying health conditions, immune response, access to healthcare, socioeconomic status, variant strains of the virus, and adherence to public health measures can impact the outcome of COVID-19 infection. While some individuals may experience mild symptoms such as fever, cough, and fatigue, others may develop severe respiratory distress and organ failure requiring intensive medical care. Additionally, some individuals may remain asymptomatic, showing no signs of illness. It is crucial to recognize and monitor these variations to better understand and manage the impacts of the pandemic and to protect vulnerable populations [6-10]. Asymptomatic infection is defined as an individual who tested positive for a laboratory-confirmed SARS-CoV-2 infection but did not exhibit any clinical symptoms associated with COVID-19 throughout the course of their illness. In contrast, pre-symptomatic infection is characterized by an individual who tested positive for SARS-CoV-2 but showed no symptoms at the time of diagnosis or during the early phase of infection but later developed COVID-19-related clinical symptoms during follow-up [7]. The first confirmed case of COVID-19 in Iraq was detected in February 2020, at a time when the government and health sector were hardly able to face such a health crisis [11]. Until April 19, 2023, Iraq witnessed four waves of COVID-19, with more than 2.4 million confirmed cases, resulting in over 25,000 deaths. About 9 million people have been vaccinated, representing approximately 25% of the Iraqi population [12]. The Iraqi government implemented several precautionary measures, including suspending in-person education in universities and schools, with some restrictions on movement since early 2020 at the onset of the first wave [12-14]. The education sector in Iraq has been plagued by various crises, including inadequate infrastructure, armed conflicts, protests, and internal migration. The COVID-19 pandemic has further exacerbated these pre-existing challenges. Consequently, the pandemic has disproportionally impacted thousands of students deprived of equitable educational opportunities due to school closure [15]. Insufficient research exists on the nature and severity of COVID-19, specifically among students in Iraq, creating a significant knowledge gap. This study aimed to fill the gap by analyzing the COVID-19 survey data from the Al-Muthanna Education Directorate to understand its impact on Iraqi school students and assess parental care of infected children during home quarantine.

## MATERIAL AND METHODS

Between November 29, 2020, and February 12, 2021, a collaborative effort was undertaken by the Department of School Environment and Health/Education Directorate in collaboration with the Public Health Directorate in Al-Muthanna Province to conduct a cross-sectional survey aimed at assessing the prevalence of COVID-19 infections among students in Al-Muthanna Province. Al-Muthanna Province, located in southwestern Iraq, covers an area of 51,740 km^2^, representing 11.9% of Iraq's total land area, and is inhabited by approximately one million residents, accounting for 3% of Iraq's overall population [16]. The province encompasses 648 schools, including 542 elementary schools, 43 intermediate schools, and 63 preparatory schools, with an overall enrollment of 253,916 students.

The selection of schools was carried out using two methods: the first method involved identifying schools with suspected cases of COVID-19 infections based on semi-daily reports from school administrators responsible for monitoring suspected cases among students. The second method entailed random selection, with schools being stratified based on type (elementary, intermediate, and preparatory schools). Subsequently, 83 schools were randomly selected from this stratification, with at least 50 students tested in each selected school.

Nine health teams, consisting of four to five well-trained health technicians, collected nasopharyngeal and oropharyngeal swabs following the COVID-19 specimen collection and processing guidelines provided by the Iraqi Ministry of Health [17]. A real-time reverse transcriptase-polymerase chain reaction (RT-PCR) assay was performed at Al-Muthanna Public Health Laboratory using the Real Line Pathogen Diagnostic Kit BI1019-96 and CFX96 Real-Time System BIO-RAD (USA). Approximately 50-150 COVID-19 tests were performed almost every day. To detect asymptomatic COVID-19 infections, swabs were randomly collected from 8,876/9,357 students who did not exhibit symptoms. For pre-symptomatic infections, students with positive COVID-19 infection were monitored for symptom onset. Students who exhibited any symptoms related to COVID-19 infection, such as fever, cough, shortness of breath or difficulty breathing, diarrhea, headache, and sore throat, were considered pre-symptomatically infected. For symptomatic infections, samples were collected from 481/9,357 students showing symptoms of illness. Students with positive COVID-19 results were home-quarantined. The RT-PCR test results typically took two or three days due to equipment and specialist shortages. Precautions were taken before and after receiving the results, including granting leave to students with symptoms until their test results returned. If the results were negative, they were permitted to return to school. Pupils with positive results were sent to home quarantine with all their classroom peers who had been in contact with them. The school was closed entirely if multiple COVID-19 cases were recorded in different classrooms. Raw data from the COVID-19 survey were obtained from the School Environment and Health Department records, including student information such as name, gender, age, presence of illness symptoms, specimen date, and home contact details. Based on this student contact information, cases of COVID-19 infection were monitored by the school administration. Parents of infected children were requested to promptly inform the school administration regarding their child's health condition, symptoms, and any new COVID-19 infections within the family.

### Statistical analysis

Raw data was collected, tabulated, and coded on a Microsoft Excel Worksheet. The statistical analysis was performed using Jamovi 2 version 2.3.28 and MedCalc Version 22.009. The odds ratio of asymptomatic, pre-symptomatic, and symptomatic COVID-19 infections in male students was calculated using female students as the reference category. A chi-square test of proportion difference was used to detect differences in the proportion of symptoms between male and female students. A chi-square test was also conducted to identify any association between symptoms and confirmed COVID-19 infection. A p-value ≤0.05 was considered statistically significant.

## RESULTS

**Figure 1:**
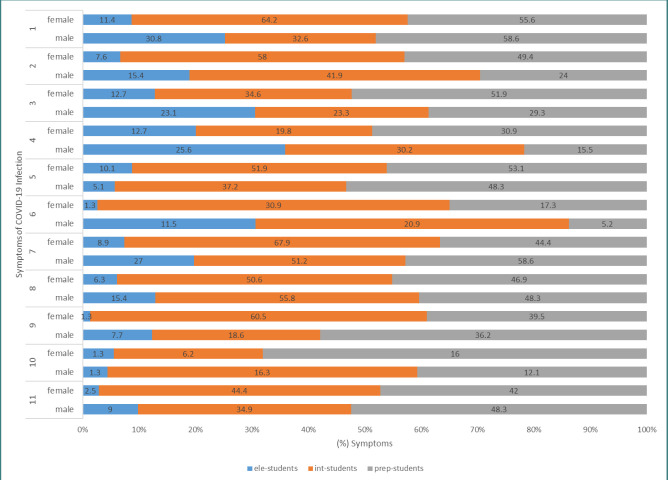
The percentage of COVID-19 symptoms in male and female elementary, intermediate, and preparatory school students. (1) fever, (2) cough, (3) headache, (4) sore throat, (5) abdominal pain, (6) sneezing, (7) diarrhea, (8) nasal congestion, (9) loss of taste and smell, (10) shortness of breath, (11) muscle/joint pain, (ele-student) elementary student, (int-student) intermediate student, (prep-student) preparatory student.

A total of 83 schools (50 elementary schools, 13 intermediate schools, and 20 preparatory schools) were visited from November 29, 2020, until the start of distance education on February 12, 2021. During this period, specialized health teams collected swabs from 9,357 students (4,261 males and 5,096 females) with a mean age of 12.67±3.63 years, representing 3.7% of the total students (Table 1). The test results confirmed the SARS-CoV-2 infection of 420 (4.5%) students, including 179/4,261 (4.2%) male students and 241/5,096 (4.73%) female students, with no significant difference in overall COVID-19 infection rates between male and female students. Among elementary students (ele-students), a total of 157 COVID-19 infections were recorded, with 78 males and 79 females affected. Within this group, 123 cases were asymptomatic (54 males and 69 females), 19 were pre-symptomatic (14 males and 5 females), and 15 were symptomatic (10 males and 5 females). The odds of asymptomatic infection were significantly lower in male ele-students compared to their female peers. Conversely, the odds of symptomatic infection were significantly higher in male ele-students compared to their female peers. Among intermediate students (int-students), 124 infections were reported, with 43 cases among males and 81 among females. This included 34 asymptomatic cases (14 males and 20 females), 50 pre-symptomatic cases (13 males and 37 females), and 40 symptomatic cases (16 males and 24 females). Furthermore, 139 infections were recorded among preparatory students (prep-students), which comprised 48 asymptomatic cases (19 males and 29 females), 68 pre-symptomatic cases (32 males and 36 females), and 23 symptomatic cases (7 males and 16 females). There were no significant differences in the odds of infection detected between elementary and preparatory male and female students (Table 2). The proportion of asymptomatic infections was 60%, with no significant difference between female and male students. Conversely, the overall proportion of infected students with symptoms was 16.2%. The odds ratio of COVID-19 infection among male students with symptoms was 2.4 times higher than that among their female counterparts with symptoms. Based on the data presented in Table 2, it can also be concluded that male int-students exhibited significantly lower odds of asymptomatic infections compared to their male elementary school peers (odds ratio [OR]=0.28, 95% confidence interval [CI]: 0.1-0.73; p=0.009). No significant difference in the odds of symptomatic infections was observed between male ele- and int-students. Similarly, the odds of asymptomatic infections in prep-students were significantly lower when compared to male ele-students (OR=0.15, 95% CI: 0.07–0.35; p<0.001). No significant difference was observed in the odds of symptomatic infections between prep- and int-students. Furthermore, there were no significant differences found in the odds of asymptomatic and symptomatic COVID-19 infections between male int-students and prep-students. Notably, the odds of asymptomatic infections in female int-students were significantly lower compared to their female peers in elementary schools (OR=0.04, 95% CI: 0.01–0.11; p<0.001). In contrast, the odds of symptomatic infections were significantly higher in female int-students (OR=5.73, 95% CI: 2.13–15.42; p<0.001) compared to their female peers in elementary schools. Similarly, the odds of asymptomatic infections were significantly lower in female prep-students (OR=0.06, 95% CI: 0.02–0.16; p<0.001) compared to their female peers in elementary schools. However, the odds of symptomatic infections in female prep-students were significantly higher (OR=7.28, 95% CI: 2.57–20.61; p<0.001) compared to their female peers in elementary schools. No significant differences were observed in the odds of asymptomatic and symptomatic COVID-19 infections between female int-students and prep-students. The study unveiled a significant association between COVID-19 infection among school students and the presence of fever, abdominal pain, diarrhea, loss of taste and smell, shortness of breath, and muscle pain. In contrast, sneezing indicated a lower likelihood of infection. There was no apparent association between the presence of cough, headache, or sore throat and COVID-19 infection. Female students manifested significantly higher occurrences of cough, abdominal pain, and loss of taste and smell than their male peers, with no significant differences in other symptoms. Shortness of breath emerged as the least prevalent symptom in both male and female students, and there were no significant differences between them, as illustrated in Table 3. Figure 1 clearly illustrates that COVID-19 symptoms manifested in varying proportions, underscoring the differential impact on male and female students. Moreover, the prevalence of each symptom may exhibit significant variation depending on the patient's age and gender. As shown in Table 4, among the 68 infected male ele-students, only 14 (20.6%) developed symptoms of COVID-19 infection. Of the 34 male ele-students with symptoms, 10/34 (29.4%) tested positive for COVID-19 infection. A chi-square test showed a significant association between infection and the presence of fever, sore throat, diarrhea, and nasal congestion. Of 74 infected female ele-students, 5/74 (6.8) developed symptoms of infection. Of 171 female ele-students with symptoms, 5/171 (2.9%) were diagnosed with infection. A significant association was detected between fever, sore throat, abdominal pain, diarrhea, and shortness of breath with positive infection. Sneezing was associated with the absence of infection. As for male int-students, among the 27 infected students, 13/27 (48.1%) exhibited pre-symptomatic infections. Moreover, among the 72 students with symptoms, 16/72 (22.2%) tested positive for the infection. Infection was significantly associated with diarrhea, nasal congestion, loss of taste and smell, some shortness of breath, and muscle/joint pain. Out of 1522 female int-students, 57 (3.75%) were diagnosed with COVID-19 infection, and only 37/57 (64.9%) developed symptoms of the infection, indicating pre-symptomatic infections among the majority of cases. Among the 94 female int-students with symptoms, 24 (25.5%) tested positive for infection. Infection was significantly associated with abdominal pain, diarrhea, nasal congestion, loss of taste and smell, some shortness of breath, and muscle/joint pain. Of 51 infected male prep-students, 32/51 (62.7%) students developed symptoms (pre-symptomatic). Among the 21 male prep-students with symptoms, seven students tested positive for COVID-19. Regarding female prep-students, out of the 65 infected students, 36/65 (55.4%) developed symptoms (pre-symptomatic). Among the 89 female prep-students with symptoms, 16 (18%) students tested positive for COVID-19. Infection was significantly associated with fever, loss of taste and smell, and some shortness of breath. Sneezing and diarrhea in female students were significantly associated with the absence of COVID-19 infections. Conversely, no significant association was observed between COVID-19 infection and symptoms such as cough, headache, sore throat, abdominal pain, and nasal congestion. Home nursing demonstrated significant efficacy in delivering appropriate care and support for children infected with COVID-19. It effectively reduced the necessity for hospitalization and minimized the risk of exposure to other infections. As documented in the medical records of the School Health Environment Department, effective home nursing encompassed activities such as symptom monitoring, administering prescribed medications, maintaining proper hygiene practices, and ensuring adequate rest and nutrition. Intermediate and preparatory school students, particularly male students, exhibited a commendable level of self-care, demonstrating effective cooperation and understanding of home quarantine procedures. These procedures encompassed hygiene practices, respiratory hygiene/cough etiquette, adherence to medical advice, maintenance of physical distancing, mask-wearing, and isolation. However, it is worth noting that the home quarantine procedures presented considerable challenges for female students across all age categories. Parents reported instances of heightened fear and anxiety among female students during the quarantine period. Importantly, there were no reported fatalities among students due to COVID-19 and no cases of severe infection, except for three students who had severe asthma.

**Table 1:** Distribution and demographics of students by school level

Visited school/Total (%)	Participants/Total(%)	Participants		Age(Mean±SD)
		Male N (%)	Female N (%)	
**Elementary** 50/542 (9.2)	4,028/171,132 (2.4)	1,813 (45)	2,215 (55)	9.2±1.16
**Intermediate** 13/43(30.2)	3,108/54,243 (5.7)	1,492 (48)	1,616 (52)	14.9±1.32
**Preparatory** 20/63(31.7)	2,221/28,541 (7.8)	956 (43)	1,265 (57)	17.6±1.29
**Total** 83/648(12.8)	9,357/253,916 (3.7)	4,261 (45.5)	5,096 (54.5)	12.67±3.63

**Table 2:** Distribution of COVID-19 cases by symptom category at different school levels

School	Without symptoms*				With symptoms**	
	Participants	Positive N (%)	Asymptomatic	Pre-symptomatic	Participants	Symptomatic Positive
**Elementary**	1,779	68 (3.8)	54/68 (79.4) †0.28, [0.09-0.82], 0.02	14/68 (20.6) 3.58, [1.12-10.55], 0.02	34	10 (29.4) 13.8, [4.36-43.94], <0.001
	2,044	74 (3.6)	69/74 (93.2)	5/74 (6.8)	171	5 (2.9)
**Intermediate**	1,420	27 (2)	14/27 (51.8) 1.99, [0.79-5.05], 0.147	13/27 (48.1) 0.5, [0.198-1.27], 0.147	72	16 (22.2) 0.83, [0.4-1.7], 0.62
	1,522	57 (3.75)	20/57 (35.1)	37/57 (64.9)	94	24 (25.5)
**Preparatory**	935	51 (5.5)	19/51 (37.3) 0.74, [0.35-1.56], 0.42	32/51 (62.7) 1.36, [0.64-2.87], 0.42	21	7 (33.3) 2.28, [0.79-6.56], 0.126
	1,176	65 (6.6)	29/65 (44.6)	36/65 (55.4)	89	16 (18)

* Without symptoms of illness at the sampling baseline

** With symptoms of illness at the sampling baseline

Cells highlighted in gray and unhighlighted cells represent male and female students, respectively. † Odds ratio, [95% confidence interval], p-value (female students are the reference categories)

**Table 3:** Comparative analysis of COVID-19 symptoms between males and females among school students

Symptoms	Total (420) / positive (%)	Male 179	Female 241	Proportion difference (%)	95% Confidence interval of proportion difference (%)	X^2^	p-value
Fever	178 (42.4)	72 (40.2)	106 (44)	3.8	-6.1-13.5	0.46	0.49
Cough	137 (32.6)	44 (24.6)	93 (38.6)	14	4.7- 22.9	8.53	0.004*
Headache	125 (29.8)	45 (25.1)	80 (33.2)	9.1	-0.02-17.9	3.68	0.055
Sore throat	93 (22.1)	42 (23.5)	51 (21.2)	2.3	-5.98-10.8	0.196	0.66
Abdominal pain	141 (33.6)	48 (26.8)	93 (38.6)	11.8	2.4-20.9	5.895	0.015*
Sneezing	61 (14.5)	21 (11.7)	40 (16.6)	4.9	-2.3-11.7	1.61	0.2
Diarrhea	175 (41.7)	77 (43)	98 (40.7)	2.3	-7.5-12.1	0.14	0.7
Nasal congestion	148 (35.2)	64 (35.8)	84 (34.9)	0.9	-8.6-10.5	0.0077	0.93
Loss of taste and smell	116 (27.6)	35 (19.6)	81 (33.6)	14	5.14-22.4	9.38	0.002*
Shortness of breath	34 (8.1)	15 (8.4)	19 (7.9)	0.5	-5.01-6.4	0.00002	0.99
Muscle/joint pain	122 (29)	50 (27.9)	72 (29.9)	2	-7.2-10.9	0.114	0.74

* significant p-value

**Table 4:** The prevalence of COVID-19 symptoms among male and female school students

School grade	COVID-19 infection	Symptoms										
		Fever	Cough	Headache	Sore throat	Abdominal pain	Sneezing	Diarrhea	Nasal congestion	Loss of taste and smell	Shortness of breath	Muscle/joint pain
**Elementary**	**Symptomatic**	^†^10/10 ^††^13/24 ^§^ 6.78 ^§§^ 0.009*	7/10 15/24 0.17 0.68	9/10 21/24 0.04 0.84	8/10 5/24 10.5 0.001*	1/10 7/24 1.44 0.23	3/10 6/24 0.09 0.76	9/10 3/24 18.6 <0.001*	6/10 2/24 10.5 0.001*	2/10 4/24 0.82 0.054	0/10 0/24 NaN NaN	4/10 15/24 1.45 0.23
		4/5 51/166 5.4 0.02*	1/5 43/166 0.09 0.77	5/5 116/166 2.13 0.15	5/5 17/166 34.9 <0.001*	5/5 27/166 22.5 <0.001*	0/5 122/166 12.8 <0.001*	5/5 75/166 5.86 0.015*	3/5 141/166 2.27 0.13	0/5 2/166 0.81 0.06	1/5 4/166 5.3 0.02*	1/5 6/166 3.32 0.07
	**Pre-symptomatic**	14/14 (100)	5/14 (35.7)	9/14 (64.3)	12/14 (85.7)	3/14 (21.4)	6/14 (42.9)	12/14 (85.7)	6/14 (42.9)	4/14 (28.6)	1/14 (7.14)	3/14 (21.43)
		5/5 (100)	5/5 (100)	5/5 (100)	5/5 (100)	3/5 (60)	1/5 (20)	2/5 (40)	2/5 (40)	1/5 (20)	0/5 (0.00)	1/5 (20)
**Intermediate**	**Symptomatic**	11/16 46/56 1.35 0.25	15/16 43/56 2.29 0.13	5/16 21/56 0.21 0.65	11/16 36/56 0.11 0.74	7/16 26/56 0.04 0.85	7/16 16/56 1.32 0.25	13/16 18/56 12.2 <0.001*	14/16 28/56 7.2 0.007*	4/16 7/56 4.9 0.03*	2/16 0/56 7.2 0.007*	8/16 9/56 5.53 0.02*
		21/24 126/139 0.23 0.632	19/24 113/139 0.06 0.81	13/24 71/139 0.04 0.81	6/24 104/139 23.2 <0.001*	16/24 42/139 9.45 0.002*	13/24 65/139 0.49 0.48	23/24 53/139 27.4 <0.001*	16/24 27/139 23.5 <0.001*	19/24 17/139 53.3 <0.001*	3/24 0/139 17.7 <0.001*	15/24 41/139 9.88 0.002*
	**Pre-symptomatic**	3/13 (23)	3/13 (23)	5/13 (38.5)	2/13 (15.4)	9/13 (69.2)	2/13 (15.4)	9/13 (69.2)	10/13 (76.9)	4/13 (30.8)	5/13 (38.5)	7/13 (53.8)
		31/37 (83.8)	28/37 (75.7)	15/37 (40.5)	10/37 (27)	26/37 (70.3)	12/37 (32.4)	32/37 (86.5)	25/37 (67.6)	29/37 (78.4)	2/37 (5.4)	21/37 (56.8)
**Preparatory**	**Symptomatic**	6/7 12/14 0.00 1	2/7 12/14 6.86 0.009*	2/7 8/14 1.53 0.22	2/7 6/14 0.4 0.53	3/7 6/14 0.00 1	0/7 11/14 11.5 <0.001*	6/7 3/14 7.88 0.005*	5/7 5/14 2.9 0.12	4/7 0/14 9.88 0.002*	4/7 1/14 6.43 0.011*	4/7 2/14 4.2 0.04*
		16/16 47/73 8.1 0.005*	13/16 62/73 0.63 0.43	11/16 61/73 1.86 0.17	8/16 33/73 0.12 0.73	11/16 63/73 0.36 0.55	5/16 51/73 8.39 0.004*	12/16 24/73 9.67 0.002*	10/16 55/73 1.1 0.29	10/16 2/73 40.2 <0.001*	8/16 2/73 29.4 <0.001*	12/16 33/40 4.66 0.03*
	**Pre-symptomatic**	28/32 (87.5)	12/32 (37.5)	15/32 (46.9)	7/32 (21.9)	25/32 (78)	3/32 (6.3)	28/32 (87.5)	23/32 (71.9)	17/32 (53)	3/32 (9.4)	24/32 (75)
		29/36 (81)	27/36 (75)	31/36 (86)	17/36 (47.2)	32/36 (88.9)	9/36 (25)	24/36 (66.7)	28/36 (77.8)	22/36 (61)	5/36 (13.9)	22/36 (61)

† Infected with symptoms/total infected, ††Not infected with symptom/total not infected, §X2, §§P value, *significant p-value Cells highlighted in darker shade represent male students; NaN: not a number; the statistical test was not performed

## Discussion

Iraq has experienced four waves of the COVID-19 pandemic, with the first wave occurring in September 2020, two subsequent waves in April and July 2021, and a fourth wave in January 2022 [12]. This study revealed the types, severity, and manifestations of COVID-19 infections among school students while also investigating the influence of gender and age on these variables. The current findings found that among 9,357 students, 420 (4.5%) tested positive for COVID-19, aligning with other studies indicating similar infection rates in school-age children [18, 19]. Analysis of daily statistics from Iraq's Ministry of Health websites revealed notable variations in cumulative infection rates across different age groups. For the years 2020, 2021, and 2022, the infection rates were 1.5%, 7.8%, 22.7%, and 24% for age groups 0-9, 10-19, 20-29, and 30-39, respectively, which clearly indicates an increase in the cumulative infection rate with age [20, 21]. Age significantly impacts infection, hospitalization, and mortality rates, with individuals aged 0-4 and 5-17 exhibiting lower rates compared to those in the 18-29 age group [22]. The present findings suggest a comparable COVID-19 infection risk among male and female students aged 12.67±3.63 years, aligning with prior studies indicating no significant gender-based differences in infection rates [23-25]. Nevertheless, other studies documented gender distinctions in school student infection risk, where female students exhibited higher COVID-19 rates compared to their male counterparts [26, 27]. Asymptomatic COVID-19 infections are cases where a person is infected with SARS-CoV-2 but exhibits no symptoms [6]. According to the current results, the ratio of asymptomatic COVID-19 infections among students aged 12.67±3.63 years was 60%, with no statistically significant difference observed between female and male students. Studies across various countries explored asymptomatic COVID-19 in 2,072 school-aged children, revealing rates exceeding 70% [24, 28-31]. Another systematic review of 18 studies found asymptomatic COVID-19 rates in children from 6.3% to 93.6%, with an overall estimate of 20.1% [19]. Among 1,152 children in 11 studies, the pooled asymptomatic rate was 27.7%, surpassing other age groups [32]. Even with comorbidities, COVID-19-infected children generally show milder symptoms. Notably, OAS1/2/3 gene cluster variations were linked to genetic predisposition for COVID-19 pneumonia in children [33]. Co-infections such as influenza and mycoplasma are among the common health problems in children during COVID-19 infection, which may exacerbate the severity of symptoms [34]. Age appears pivotal in asymptomatic infection rates, with older adults, particularly males, showing more severe symptoms than young adults [35]. In addition to age and body weight, COVID-19 risk encompasses employment status and place of residence [36]. A pooled analysis of 95 studies with a sample size of over 29 million individuals revealed that 40.5% of those who tested positive for COVID-19 were asymptomatic [37], which poses a significant risk factor for increased community transmission. The present findings suggest that fever, abdominal pain, diarrhea, loss of taste and smell, shortness of breath, and muscle pain emerged as notable indicators of COVID-19 infection. In 2020, a study in Sulaimaniyah City, Iraq, identified fever as the predominant symptom among children with COVID-19 [38]. In line with Pérez-Gaxiola *et al*., the present study revealed that apart from diarrhea and nasal congestion, fever, cough, and headache stood out as some of the most prevalent symptoms among infected males and females [39]. Age can significantly influence the severity and the specific manifestations of COVID-19 symptoms [40]. It is evident that relying on COVID-19 symptoms observed in adults is not reliable for diagnosing infection in children [41, 42]. The current findings indicate that diarrhea and abdominal pain, unusual for respiratory infections, are strongly linked to COVID-19 infection in children. This aligns with Zimmermann and Curtis, who noted similarities between COVID-19 and gastrointestinal infections in children [43]. The current findings highlight the need for increased awareness and consideration of atypical symptoms when screening and testing for COVID-19 in children. Recognizing the association between diarrhea, abdominal pain, and COVID-19 infection may lead to earlier identification and isolation of infected children, which could reduce transmission and improve outcomes. On the other hand, the study found that the presence of sneezing was associated with a lower likelihood of COVID-19 infection, which may be surprising given that it is a common symptom of respiratory infections. The current study did not detect any noticeable association between COVID-19 infection and the existence of cough, headache, or sore throat. Other clinical criteria can be adopted to track the course of COVID-19 infections in hospitalized children, as Henry *et al*. recommended. Pediatricians should serially monitor c-reactive protein (CRP), procalcitonin (PCT), and lactate dehydrogenase (LDH). The authors also concluded that elevated creatine kinase-MB (CK-MB) in mild pediatric COVID-19 cases is indicative of possible cardiac injury [44]. Lymphopenia is less common, and elevated creatine kinase-MB (CK-MB) is more common in children with COVID-19 compared to adults, suggesting that heart injury would be more likely to occur in pediatric patients [45]. The refusal to accept the COVID-19 vaccine has significantly contributed to the ongoing challenges in COVID-19 management. A study at Al-Zahraa University in Iraq found that 70.4% of students rejected the COVID-19 vaccine, with concerns about safety and a lack of awareness about the different types of vaccines [46]. Furthermore, the COVID-19 pandemic caused a reduction in the vaccination coverage rate (VCR) among children in Iraq, indicating the need for new health policies that can help increase VCR [47]. Implementing measures that restrict mobility can potentially have a modest impact on slowing down the spread of COVID-19. Therefore, it is recommended to complement mobility-limiting measures with other public health interventions, such as ubiquitous mask usage [13]. Considering the outcomes gleaned from this study, extending home-based healthcare to children, involving interventions like administering antipyretics, ensuring optimal ventilation, maintaining proper hydration, and employing pain-relief medications, emerges as a viable strategy for tackling COVID-19. In Iraq, schools have been closed repeatedly during the last two years due to the COVID-19 pandemic forcing a shift to online learning. The choice to temporarily close schools in Iraq for extended periods might indeed be a sound decision. Research conducted by Chernozhukov *et al*. suggests that regions that opted for in-person K-12 schooling experienced, on average, a 5-percentage point increase in the rate of COVID-19 case growth [48]. Improving e-learning should not only focus on technical improvements, such as enhancing the quality of the internet and the devices used. Instead, it should also encompass rehabilitation programs for children to enhance their cyberculture. This approach can assist children in avoiding harmful websites, addressing the concerns of mothers who worry about the lack of control over the quality of information their children access through e-learning [39]. In addition to implementing home nursing procedures, it is essential to provide children with emotional support, as they might grapple with feelings of fear or anxiety while undergoing isolation and quarantine [49]. The present study stands as the sole of its kind in Iraq, but it did not explore the cons and pros of school closures or dive into how community infection rates interact with student infections or vice versa. These findings emphasize the necessity for immediate evidence-based interventions to contain COVID-19 transmission among students. Multiple visits to the same school were not feasible due to limitations in human resources and technical capabilities. Tracking new COVID-19 cases for educator-to-educator, educator-to-student, student-to-student transmission was challenging. Self-reported symptoms might carry recall and social biases. Symptoms were assessed by parents and school staff rather than healthcare professionals. However, this approach introduces a potential for inaccuracies in gauging both the nature and severity of symptoms. No blood tests were adopted to assess different aspects of the COVID-19 infection (e.g., complete blood count (CBC), C-reactive protein (CRP), D-dimer, Ferritin, and Interleukin-6 (IL-6)). Many sociodemographic factors, such as ethnicity/race, socioeconomic status, geographic location, comorbidities, and living conditions, were not included in the study nor analyzed.

## CONCLUSION

Asymptomatic COVID-19 infections were more prevalent than symptomatic infections among school students with a mean age of 12.67±3.63 years. Male students exhibited a higher likelihood of testing positive for COVID-19 compared to female peers upon symptom onset, with students aged 9.2±1.16 years showing higher rates of asymptomatic infections than their older counterparts. Fever, abdominal pain, diarrhea, loss of taste and smell, shortness of breath, and muscle pain were strongly linked to COVID-19 infection. Sneezing was associated with the absence of COVID-19 infection. There may be gender- and age-related differences in the manifestation of symptoms.

## References

[1] Rajapakse N, Dixit D (2021). Human and novel coronavirus infections in children: a review. Paediatr Int Child Health.

[2] Zhou F, Yu T, Du R, Fan G (2020). Clinical course and risk factors for mortality of adult inpatients with COVID-19 in Wuhan, China: a retrospective cohort study. Lancet.

[3] Barber RM, Sorensen RJ, Pigott DM, Bisignano C (2022). Estimating global, regional, and national daily and cumulative infections with SARS-CoV-2 through Nov 14, 2021: a statistical analysis. Lancet.

[4] Ioannidis JPA (2021). Reconciling estimates of global spread and infection fatality rates of COVID-19: An overview of systematic evaluations. Eur J Clin Invest.

[5] Sorensen RJ, Barber RM, Pigott DM, Carter A (2022). Variation in the COVID-19 infection-fatality ratio by age, time, and geography during the pre-vaccine era: A systematic analysis. Lancet.

[6] Cui X, Zhao Z, Zhang T, Guo W (2021). A systematic review and meta-analysis of children with coronavirus disease 2019 (COVID-19). J Med Virol.

[7] Gao W, Lv J, Pang Y, Li LM (2021). Role of asymptomatic and pre-symptomatic infections in covid-19 pandemic. BMJ.

[8] Meintrup D, Nowak-Machen M, Borgmann S (2021). Nine Months of COVID-19 Pandemic in Europe: A Comparative Time Series Analysis of Cases and Fatalities in 35 Countries. Int J Environ Res Public Health.

[9] Velicu MA, Furlanetti L, Jung J, Ashkan K (2021). Epidemiological trends in COVID-19 pandemic: prospective critical appraisal of observations from six countries in Europe and the USA. BMJ Open.

[10] Bollyky TJ, Hulland EN, Barber RM, Collins JK (2022). Pandemic preparedness and COVID-19: an exploratory analysis of infection and fatality rates, and contextual factors associated with preparedness in 177 countries, from Jan 1, 2020, to Sept 30, 2021. Lancet.

[11] Hantoosh SM The prevalence of side-effects after BNT162B2/Pfizer, AZD1222/Astra Zeneca, and BBIBP-CorV/Sinopharm vaccines among Iraqi residents. MINAR CONGRESS.

[12] World Health Organization COVID-19 Dashboard: Iraq. https://covid19.who.int/region/emro/country/iq.

[13] Lami F, Khaleel HA, Khader YS (2021). Mobility indicators and COVID-19 growth ratio in Iraq: a correlation study. J Public Health (Oxf).

[14] Aldulaimi MH, Kadhim TA, Al-Nidawi WJ, Kzar MH (2023). Covid-19 pandemic effects on the sustainable development of learning in Iraq. AIP Conference Proceedings.

[15] Shamseldin N (2020). Education in Iraq–Impact of COVID-19, protests and pre-existing crises on needs overview. ACAPS.

[16] Central Statistical Organization in Iraq (2020). Population Estimates in Iraq. Central Statistical Organization in Iraq.

[17] Petruzzi G, De Virgilio A, Pichi B, Mazzola F (2020). COVID-19: Nasal and oropharyngeal swab. Head Neck.

[18] Yung CF, Kam KQ, Chong CY, Nadua KD (2020). Household transmission of severe acute respiratory syndrome coronavirus 2 from adults to children. J Pediatr.

[19] Gudbjartsson DF, Helgason A, Jonsson H, Magnusson OT (2020). Spread of SARS-CoV-2 in the Icelandic Population. N Engl J Med.

[20] Kurdistan Regional Government Ministry of Health. Ministry of Health.

[21] Ministry of Health Iraq. https://www.facebook.com/MOH.GOV.IQ.

[22] Centers for Disease Control and Prevention COVID-19 Hospitalization and Death by Age. https://www.cdc.gov/coronavirus/2019-ncov/covid-data/investigations-discovery/hospitalization-death-by-age.html.

[23] Zimmerman KO, Akinboyo IC, Brookhart MA, Boutzoukas AE (2021). Incidence and secondary transmission of SARS-CoV-2 infections in schools. Pediatrics.

[24] Dong Y, Mo X, Hu Y, Qi X (2020). Epidemiology of COVID-19 Among Children in China. Pediatrics.

[25] Chang TH, Wu JL, Chang LY, Kuo CH (2021). Characteristics and outcomes of hospitalized children and adolescents with COVID-19 in New York City. J Formos Med Assoc.

[26] Alqahtani JS, Alfelali M, Alkhathlan EA, Alqahtani AS (2021). Epidemiology of COVID-19 among school-aged children in Saudi Arabia: A retrospective study. J Infect Public Health.

[27] Kim YE, Kim S, Chang JH, Lee H (2021). High school students are more vulnerable to COVID-19 than adults: A Korean nationwide study. PLoS One.

[28] Liguoro I, Pilotto C, Bonanni M, Ferrari ME (2020). SARS-COV-2 infection in children and newborns: a systematic review. Eur J Pediatr.

[29] Burke RM, Killerby ME, Newton S, Ashworth CE (2020). Symptom Profiles of a Convenience Sample of Patients with COVID-19-United States, January-April 2020. MMWR Morb Mortal Wkly Rep.

[30] Pérez-Gaxiola G, Flores-Rocha R, Valadez-Vidarte JC, Hernández-Alcaraz M (2021). Clinical and epidemiological characteristics of children with SARS-CoV-2 infection: a case series in Sinaloa. Bol Med Hosp Infant Mex.

[31] Martins MM, Prata-Barbosa A, da Cunha AJLA (2021). Update on SARS-CoV-2 infection in children. Paediatr Int Child Health.

[32] He J, Guo Y, Mao R, Zhang J (2021). Proportion of asymptomatic coronavirus disease 2019: A systematic review and meta-analysis. J Med Virol.

[33] Di Pietro GM, Ronzoni L, Meschia LM, Tagliabue C (2023). SARS-CoV-2 infection in children: A 24 months experience with focus on risk factors in a pediatric tertiary care hospital in Milan, Italy. Front Pediatr.

[34] Zimmermann P, Curtis N (2020). COVID-19 in Children, Pregnancy and Neonates: A Review of Epidemiologic and Clinical Features. Pediatr Infect Dis J.

[35] Ali HN, Ali KM, Rostam HM, Ali AM (2022). Clinical laboratory parameters and comorbidities associated with severity of coronavirus disease 2019 (COVID-19) in Kurdistan Region of Iraq. Pract Lab Med.

[36] Jouda J, Abdul Kareem Jabbar E, Salih Abdulhadi F, Atiyah Kamil Y (2022). Assessment of some Physiological Biomarkers in COVID-19 Patients in Thi-Qar, Iraq. Arch Razi Inst.

[37] Ma Q, Liu J, Liu Q, Kang L (2021). Global Percentage of Asymptomatic SARS-CoV-2 Infections Among the Tested Population and Individuals With Confirmed COVID-19 Diagnosis: A Systematic Review and Meta-analysis. JAMA Netw Open.

[38] Salih AF, Hamasalih K, Rahman HS, Mohammed GA (2022). Pediatric COVID-19 infection in Sulaimaniyah Governorate, Iraq. Am J Otolaryngol.

[39] Widiasih R, Suryani S, Rakhmawati W, Arifin H (2022). The Impact of Online Learning among Adolescents during the COVID-19 Pandemic: A Qualitative Study of Mothers' Perspectives. Iran J Nurs Midwifery Res.

[40] Stokes EK, Zambrano LD, Anderson KN, Marder EP (2020). Coronavirus Disease 2019 Case Surveillance-United States, January 22-May 30, 2020. MMWR Morb Mortal Wkly Rep.

[41] Pérez-Gaxiola G, Flores-Rocha R, Valadez-Vidarte JC, Hernández-Alcaraz M (2021). Clinical and epidemiological characteristics of children with SARS-CoV-2 infection: a case series in Sinaloa. Bol Med Hosp Infant Mex.

[42] De Souza TH, Nadal JA, Nogueira RJ, Pereira RM, Brandão MB (2020). Clinical manifestations of children with COVID-19: a systematic review. Pediatr Pulmonol.

[43] Zimmermann P, Curtis N (2020). Coronavirus infections in children including COVID-19: an overview of the epidemiology, clinical features, diagnosis, treatment and prevention options in children. Pediatr Infect Dis J.

[44] Henry BM, Benoit SW, de Oliveira MHS, Hsieh WC (2020). Laboratory abnormalities in children with mild and severe coronavirus disease 2019 (COVID-19): a pooled analysis and review. Clin Biochem.

[45] Cui X, Zhang T, Zheng J, Zhang J (2020). Children with coronavirus disease 2019: a review of demographic, clinical, laboratory, and imaging features in pediatric patients. J Med Virol.

[46] Hadi Al Kazzaz H (2021). COVID19 vaccination choice among Iraqi students at Al-Zahraa University for women. F1000Res.

[47] Alhaddad AR, Ahmadnezhad E, Fotouhi A (2022). The vaccination coverage rate in under-5 children in Nasiriyah, Iraq before and during the COVID-19 pandemic. Epidemiol Health.

[48] Chernozhukov V, Kasahara H, Schrimpf P (2021). The association of opening K–12 schools with the spread of COVID-19 in the United States: County-level panel data analysis. Proc Natl Acad Sci U S A.

[49] Camacho-Montaño LR, Iranzo A, Martínez-Piédrola RM, Camacho-Montaño LM (2022). Effects of COVID-19 home confinement on sleep in children: A systematic review. Sleep Med Rev.

